# Clinical outcomes with a new diffractive multifocal intraocular lens optimized by the dynamic light utilization algorithm

**DOI:** 10.1038/s41433-024-03435-0

**Published:** 2024-11-06

**Authors:** Jorge L. Alió, Elinor Megiddo Barnir, Ronald Steven S. Medalle, Ana B. Plaza-Puche, Antonio Martínez, Pilar Yébana, Blanca Poyales, Francisco Poyales

**Affiliations:** 1 Vissum Grupo Miranza, Alicante, Spain; 2 Division of Ophthalmology, Universidad Miguel Hernández, Alicante, Spain; 3 Ophthalmology Department, The Edith Wolfson Medical Center, Holon, Israel; 4 Sackler Faculty of Medicine Tel Aviv University, Tel Aviv, Israel; 5 Cornea and Refractive Service, Associated Cebu Eye Specialists, Cebu, Philippines; 6 Department of Ophthalmology, Cebu Institute of Medicine, Cebu, Philippines; 7 Miranza IOA, Madrid, Spain

**Keywords:** Medical research, Diseases, Eye diseases

## Abstract

**Background/objectives:**

To evaluate the refractive outcomes, optical performance, and the quality of vision in patients implanted with a new diffractive intraocular lens (IOL), the Intensity Hanita.

**Subjects/methods:**

This observational, prospective, longitudinal study included 64 eyes underwent bilateral cataract surgery with the Intensity IOL (Hanita Israel) implantation. Main outcome measures after 6 months were the following visual acuities (VAs) of uncorrected and corrected distance (UDVA and CDVA), uncorrected and distance corrected intermediate VAs (UIVA and DCIVA), uncorrected and distance corrected near (UNVA and DCNVA), refraction, slitlamp biomicroscopy, defocus curve (DFC), high ocular aberrations (HOA), contrast sensitivity (CS), optical quality, subjective quality of vision (QoV) and near activity visual questionnaires (NAVQ).

**Results:**

Sixty-six percent of eyes having UDVA 0.10 logMAR or better. DFC showed maximum vision at distance (0.02 ± 0.07 LogMAR at 0.0 D), with flat decline through intermediate and near vision (0.11 ± 0.08 LogMAR at −1.5 D and 0.12 ± 0.12 at −2.5 D). No significant changes in CS were found (all spatial frequencies, *p* ≥ 0.06). The RMS of HOA, coma, trefoil, and SA were 0.21 ± 0.10, 0.10 ± 0.06, 0.11 ± 0.07, and 0.00 ± 0.04 μm and the Strehl ratio was 0.12 ± .04 at 6 months. Subjective symptoms (halos and glare) were reported mild but well tolerated, not causing significant disturbance in daily activities. The NAVQ showed high levels of satisfaction performing daily near-vision tasks.

**Conclusions:**

The Hanita Intensity diffractive IOL successfully restores all distances of vision. The flat profile of the monocular defocus curve confirms the five-foci distribution principle that provides vision at all ranges while increasing the depth of focus.

## Introduction

Within the last few decades, cataract surgery has evolved into a refractive procedure due to a growing demand to achieve spectacle independence across all ranges of vision. With the introduction of premium intraocular lenses, the achievement of spectacle independence has met this demand to a good extent. These lenses have included multifocal (mfIOL), extended depth of focus lenses as well as the toric versions of both types, all with the aim to provide optimal vision at various distances [1–4].

While mfIOLs have provided adequate visual acuity (VA) at different distances, allowing patients to be independent from glasses, they also come with their own side effects due to the various ways by which these lenses bend, distribute, and focus light as a consequence of their optical design to produce multifocality. These side effects include artifacts produced by the mentioned mechanisms which can cause some discomfort or distortions of vision such as glares or haloes and other side effects include decreased contrast sensitivity (CS), and positive and negative dysphotopsias [5–9]. These effects are also related to the optical design of the lens and can cause dissatisfaction among patients, even leading to the need for lens explantation [10, 11]. Companies have been designing IOLs in such a way to lessen these side effects while still providing good retinal image quality.

The new Hanita Intensity SL [12] diffractive multifocal IOL (Hanita Lenses Ltd, Kibbutz Hanita, Israel) addresses these issues by its design, the Dynamic Light Utilization algorithm, which maximize the light efficiency. The energy distribution was established with the goal of achieving a smooth transition between visual ranges and increased visual function for near range providing independence from spectacles.

The aim of this study is to evaluate the visual, refractive, and optical quality outcomes as well as patient-reported outcome measures (PROMs) of patients after cataract surgery with the Intensity diffractive IOL implantation.

## Materials/subjects and methods

### Study design

This was an observational, prospective, multicentric, longitudinal, study involving patients eligible for cataract surgery with the implantation of the Intensity lens. The study was conducted by the Cornea, Cataract, and Refractive Surgery Unit in Vissum Alicante, and Miranza IOA Madrid, Grupo Miranza, Spain. Preoperative and postoperative assessments were performed at five intervals: preoperatively, on the first day after surgery, then on the first, third, and sixth month after. All patients were adequately informed and signed a consent form. The study adhered to the tenets of the Declaration of Helsinki (2013 Revision) and was approved by the Instituto de microcirugía ocular Grupo Miranza Ethical Board Committee.

### Patients

The study population comprised 64 eyes of 32 bilateral cataract surgery patients, aged 50–79 years (mean 66.71 ± 8.83 years), who were implanted with the Hanita Intensity SL diffractive IOL, Hanita Lenses Ltd, Hanita, Israel. The inclusion criteria of this study were as follows: patients with bilateral visual significant cataracts interested in bilateral cataract surgery, 50–80 years of age, and calculated IOL power within the available diopter range of 10–30 D (in 0.50 D increments). The exclusion criteria were comorbidities potentially causing postoperative visual impairment such as degenerative macular pathology and other diseases, congenital ocular anomalies, dry eye, and reading disabilities. Two patients lost the follow-up visit at 6 months after implantation and the data of these patients were only analyzed up to 3 months of follow-up.

### Preoperative examination

Preoperatively, all patients had a full ophthalmologic examination including the evaluation of the refractive status, the distance and near visual acuities, slit lamp examination, applanation tonometry, retinal OCT, and funduscopy. Distance and near VA were measured with the early treatment diabetic retinopathy study (ETDRS) reading chart and the Radner reading chart, respectively. Other examinations included corneal topography (MS39 AS-OCT, Costruzione Strumenti Oftalmici (CSO), Firenze, Italy) and biometry (IOL master 700, Carl Zeiss Meditec, Germany) IOL power calculation was performed with optical coherence interferometry using the Barrett Universal II formula version 1.05. The target refraction was emmetropia in all cases that were included.

### Surgery

Surgeries were performed at Vissum Alicante and Miranza IOA Madrid (Miranza group) using the standard technique of phacoemulsification. All patients received topical anesthesia before surgery. Adequate dilatation was obtained with intracameral mydriatics. The main corneal incision of 2.2 mm was placed on the axis of the positive corneal meridian. Postoperative topical therapy included a combination of topical antibiotics and steroid agents (Tobradex, Alcon, Barcelona).

### The IOL

The Intensity SL IOL [12] (Hanita Lenses Ltd, Kibbutz Hanita, Israel) is a new diffractive multifocal IOL that consists of an anterior spherical surface and posterior aspheric-diffractive design, divided into zones for pupil aperture optimization. This lens is a single-piece IOL with an optic diameter of 6.0 mm, and an overall length of 13.0 mm with a 360° continuous square edge optic (Supplementary material Fig. A). The lens has a five-foci design with additions of +1.50 and +3.00 D in the IOL plane for intermediate and near, and two additional diffractive orders providing the lens with a greater efficacy. These two additional foci between the distance and intermediate foci, as well as between the intermediate and near are to enable a smooth transition through a wide range of distances. This IOL is made from hydrophilic acrylic material with a bonded UV absorber and violet light filter. It has an open C-loop haptic design with a 5° haptic angulation and spherical aberration of −0.13 μm.

### Postoperative examination

Patients were evaluated during the follow-up at 1 day, 1 month, 3 months, and 6 months after surgery by experienced optometrists certified in Good Clinical Practice. Distance-corrected near visual acuity (DCNVA) and the intermediate at 63 cm (DCIVA) visual acuities were only measured during the postoperative period, using the ETDRS chart. The postoperative examination protocol at 1, 3, and 6 months was identical to the preoperative with additional measurement of the contrast sensitivity function (CSF) in mesopic conditions (CSV 1000, VectorVision Ocular Health, Greenville, Ohio, USA) at 1 and 6 months. Ocular aberrometry (Osiris, CSO, Firenze, Italy) with a 4.0 mm pupil size was measured at 3 and 6 months. The second-order aberrations were excluded to avoid bias caused by residual astigmatism in the PSF Strehl ratio measurement; the resulting parameter is the PSF Strehl ratio (PSFw2). The defocus curves at 6 months were also recorded. The defocus curves were obtained in monocular and binocular conditions and with best distance correction by adding plus lenses in 0.50 D increments and recording the VA achieved by the patient with each step of blur. The test was repeated but with negative lenses.

At 3 months follow-up, additional examinations were performed for PROMs; the Quality of Vision (QoV) and the Near Activity Visual Questionnaire (NAVQ-10) questionnaires.

### Statistical analysis

The statistical analysis was performed with the SPSS statistical software package version 18.0 for Windows (SPSS, Inc) and R software. The average values and standard deviations were calculated for every parameter during the follow-up. A non-parametric statistical test, the Wilcoxon signed rank test [13], made comparisons of clustered data to adjust for any correlation between fellow eyes of the same patient, all eyes were included to increase the power of comparisons. This test was applied to assess the significance of differences between preoperative and postoperative data, using in all cases the same level of significance (*p* < 0.05).

## Results

### Visual and refractive outcomes

**Fig. 1:**
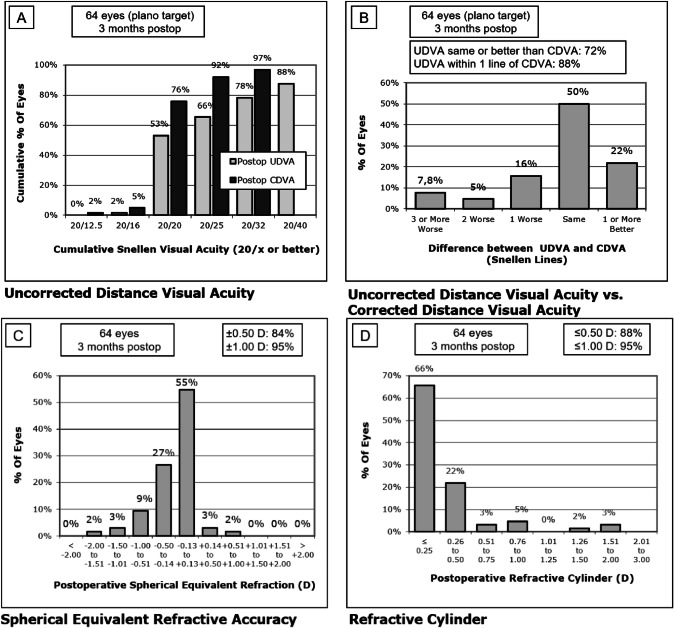
Standard graphs for reporting refractive outcomes after IOL implantation in cataract surgery obtained after 3 months of Intensity IOL implantation.

Figure 1 shows the standard graphs for reporting refractive outcomes after IOL implantation. Of all the patients, 66% eyes achieved uncorrected distance visual acuity (UDVA) of 0.10 logMAR or better. The UDVA outcomes are same or better than the CDVA for 72% of eyes. And the 88% of eyes shows a UDVA within one line of CDVA.

Table 1 summarizes the pre- and post-operative visual conditions of the eyes. At 1 month after surgery, a statistically significant improvement was observed in the UDVA and uncorrected near visual acuity (UNVA), (Wilcoxon test, all *p* < 0.01). No significant changes in these visual parameters were observed in the remaining follow-up periods (Wilcoxon test, *p* ≥ 0.05). No significant change in DCNVA was detected between 1 and 3 months after surgery (Wilcoxon test, *p* = 0.88), but a significant improvement was found between 3 and 6 months after surgery (Wilcoxon test, *p* < 0.01). No significant changes in visual intermediate parameters were observed during the postoperative follow-up (Wilcoxon test, *p* ≥ 0.31).

**Table 1:** Comparative table shows the preoperative and postoperative visual conditions of patients included in this study.

Mean (SD) range	Preop	1 month	3 months	6 months	*P* value pre-1 month
UDVA (LogMAR)	0.54 (0.32) 0–1.09	0.12 (0.15) −0.04 to 0.69	0.13 (0.18) −0.07 to 0.69	0.11 (0.15) −0.04 to 0.74	<0.01
Sphere (D)	0.57 (2.28) −6.0 to 4.0	−0.09 (0.4) −1.5 to 1.0	−0.01 (0.46) −1.25 to 1.25	−0.01 (0.51) −2.0 to 1.25	0.02
Cylinder (D)	−0.66 (0.62) −2.00 to 0.25	−0.29 (0.46) −2.0 to 0.0	−0.30 (0.47) −2.0 to 0.0	−0.36 (0.5) −2.0 to 0.0	<0.01
CDVA (LogMAR)	0.10 (0.17) −0.07 to 0.76	0.05 (0.08) −0.07 to 0.3	0.04 (0.09) −0.07 to 0.37	0.02 (0.06) −0.08 to 0.3	0.06
UNVA (LogMAR)	0.47 (0.2) 0.22–0.79	0.11 (0.13) −0.10 to 0.48	0.18 (0.13) 0.0–0.58	0.13 (0.1) 0.0–0.39	<0.01
DCNVA (LogMAR)	–	0.12 ± 0.12 −0.08 to 0.52	0.16 ± 0.12 0.00–0.58	0.12 ± 0.09 0.00–0.30	
UIVA (LogMAR)	–	0.10 ± 0.16 −0.16 to 0.50	0.15 ± 0.14 0.00–0.60	0.13 ± 0.12 −0.10 to 0.40	
DCIVA (LogMAR)	–	0.08 ± 0.14 −0.16 to 0.50	0.15 ± 0.14 0.00–0.60	0.13 ± 0.12 −0.10 to 0.40	
CNVA (LogMAR)	0.13 (0.18) 0.0–0.69	0.09 (0.07) 0.0–0.22	0.11 (0.12) 0.0–0.58	0.08 (0.06) 0.0–0.22	0.71
Addition	2.61 (0.28) 2.0–3.0	0.40 (0.39) 0.0–1.5	0.36 (0.37) 0.0–1.0	0.32 (0.41) 0.0–1.5	<0.01

The corresponding *p* values for the comparison between preoperative and postoperative follow-up are shown for each parameter evaluated.

*SD* standard deviation, *D* dioptres, *UDVA* uncorrected distance visual acuity, *CDVA* corrected distance visual acuity, *UNVA* uncorrected near visual acuity, *DCNVA* distance corrected near visual acuity, *UIVA* uncorrected intermediate visual acuity, *DCIVA* distance corrected intermediate visual acuity, *CNVA* corrected near visual acuity.

Regarding manifest refraction, significant changes were found in the sphere and cylinder 1 month after surgery (Wilcoxon test, *p* < 0.02), with no significant modifications afterwards (Wilcoxon test, *p* ≥ 0.05) (Table 1). Eighty-four percent and 95% of eyes are within a spherical equivalent of ±0.50 and ±1.00 D, respectively (Fig. 1).

### Defocus curve

**Fig. 2:**
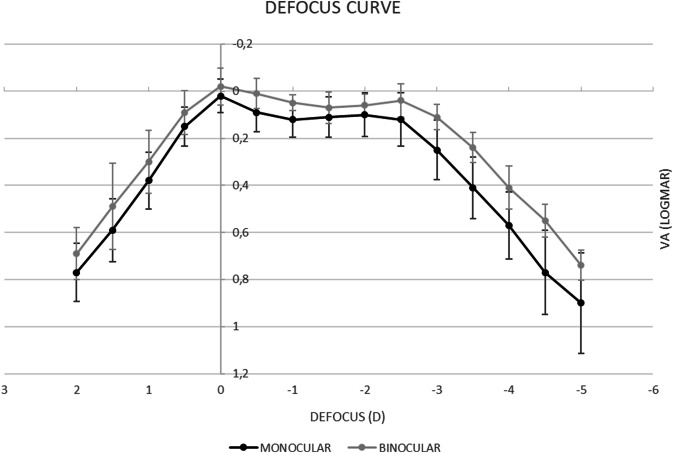
Mean monocular and binocular defocus curve of patients implanted with the Intensity MF IOL.

Figure 2 shows the mean monocular and binocular defocus curve of the patients analyzed in the current study, respectively. It was found that this multifocal IOL provided a relatively flat profile showing peak of maximum vision at distance (around 0.00 D defocus level), with a flat decline through the intermediate and near vision.

### Contrast sensitivity function

Supplementary material Fig. B shows the mean postoperative CSF in logarithmic scale under mesopic conditions at 1 and 6 months after surgery. At 1 month mean CS for the spatial frequencies of 3, 6, 12, and 18 cycles per degree (CPD) were 1.53 ± 0.19, 1.75 ± 0.22, 1.47 ± 0.28, and 1.07 ± 0.25 log units, respectively. At 6 months mean CS for the spatial frequencies of 3, 6, 12, and 18 CPD were 1.58 ± 0.14, 1.90 ± 0.18, 1.60 ± 0.30, and 123 ± 0.32 log units, respectively. No significant changes in CSF were observed for all spatial frequencies (Wilcoxon test, *p* ≥ 0. 31).

### Retinal optical quality assessment

At 3 and 6 months after surgery, there was no significant change for the root mean square (RMS) of the high order aberrations (HOA) (0.24 ± 0.12–0.21 ± 0.10 µm), the trefoil (0.11 ± 0.08–0.11 ± 0.07 µm), coma (0.10 ± 0.05–0.10 ± 0.06 µm) and spherical aberrations (0.00 ± 0.09–0.00 ± 0.04 µm) (Wilcoxon test, *p* ≥ 0.05). Regarding the optical quality analysis for far vision, no significant change of the ocular mean PSF Strehl ratio (PSFw2) was observed from 0.12 ± 0.05 at 3 months to 0.13 ± 0.05 at 6 months after surgery (Wilcoxon test, *p* ≥ 0.05).

### PROMs: quality of vision and quality of life outcomes

Tables 2 and 3 summarize the achieved mean QoV and life outcomes in patients implanted with the Intensity MF IOL. Patients had more frequent haloes, though well tolerated by the patients. Regarding life quality of near vision, patients had more difficulty seeing close objects in poor or dim light and maintaining focus for prolonged near work. Over 90% of patients were completely or very satisfied with their near vision (Tables 2 and 3).

**Table 2:** Mean values of the QoV questionnaire items at 3 months postoperatively.

Item	Frequency	SD	Severity	SD	Bothersome	SD
Glare	0.84	1.08	0.94	1.01	0.84	1.02
Haloes	2.28	1.02	1.91	0.82	1.28	0.85
Starbursts	1.13	1.34	0.94	1.08	0.78	1.04
Hazy vision	1.06	1.16	0.88	0.87	0.97	1.03
Blurred vision	0.88	1.16	0.78	0.97	0.78	1.07
Distortion	0.19	0.59	0.19	0.59	0.16	0.57
Double or multiple images	0.09	0.30	0.09	0.30	0.06	0.25
Fluctuation in vision	0.41	0.80	0.39	0.67	0.42	0.81
Focusing difficulties	0.32	0.65	0.35	0.71	0.32	0.65
Depth perception difficulties	0.23	0.67	0.19	0.54	0.16	0.45

Grading scale: 0, never/not at all/ not at all; 1, occasionally/mild/little; 2, quite often/moderate/quite; 3, very often/sever/very.

**Table 3:** Mean values of the NAVQ-10 questionnaire items at 3 months postoperatively.

Item	Punctuation	SD
1. Reading small print, such as: newspaper articles, items on a menu, telephone directories	0.30	0.68
2. Reading labels/instructions/ingredients/prices such as on: medicine bottles, food packing	0.39	0.79
3. Reading post/mail, such as: electric bill, greeting cards, bank statements, letters from friends and family	0.09	0.52
4. Writing and reading own writing, such as: greeting cards, notes, letters, filling in forms, checks, signing own name	0.09	0.52
5. Seeing the display and keyboard on a computer or calculator	0.24	0.61
6. Seeing the display and keyboard on a mobile or fixed telephone	0.21	0.60
7. Seeing objects close to you and engaging in your hobbies, such as: playing card games, gardening, seeing photographs	0.47	0.76
8. Seeing objects close to you in poor or dim light	0.88	0.99
9. Maintaining focus for prolonged near work	0.67	0.89
10. Conducting near work	0.45	0.75
Overall near vision satisfaction	0.97	1.05

Grading scale for items 1–10: 0, no difficulty; 1, a little difficulty; 2, moderate difficulty; 3, extreme difficulty. Grading scale for overall satisfaction: 0, completely satisfied; 1, very satisfied; 2, moderately satisfied; 3, a little satisfied; 4, completely satisfied.

### Surgical and postoperative complications

No postoperative complications were observed, no significant posterior capsule opacification causing visual decrease of 1 or more lines associated with visual symptoms and leading to neodymium-doped yttrium aluminum garnet (Nd:YAG) laser capsulotomy occurred during the follow-up period. No significant IOL decentration was detected at the slit lamp examination either.

## Discussion

MfIOL designs have improved over the years causing less photic phenomena while maintaining the today’s high standard the ability to have a good range of VA of different distances. MfIOLs have caused inconvenience by producing undesirable symptoms in some cases, such as the presence of photic phenomena, and the reduction of the CSF [11, 14–16]. This IOL was designed to have efficient light energy distribution that was made possible by the modified algorithm (dynamic light distribution), resulting in a lens profile different than most on the market today [12].

After one month from implantation of the IOL, the UDVA, UNVA, CDNVA, and subjective refraction had already been achieved, which were successful in outcomes, and all stabilized throughout the rest of the follow-up period. The IOL was able to restore the visual function after cataract surgery. When our results were compared to other publications, the VA outcomes match that of data published especially that of multifocal IOLs [17–24]. The mean UDVA achieved with the Intensity IOL at the end of follow-up was. 0.11 ± 0.15 logMAR, compared to the one achieved by the TECNIS Monofocal ZCB00 in a study, the post operative mean was 0.01 ± 0.13 logMAR. As for near and intermediate vision, the achieved means of 0.13 ± 0.1 logMAR and 0.15 ± 0.13 logMAR, respectively. Our results achieved better mean VA than the TECNIS which resulted in a UIVA of 0.33 ± 0.19 logMAR and a UNVA of 0.61 ± 0.18 logMAR. Regarding percentages and compared to monofocal IOLs, the Cumulative UDVA of 0.1 logMAR or better was achieved in 66% of the eyes implanted with the Intensity, compared to the TECNIS Monofocal ZBCB00 which achieved this VA in 95.3% of eyes implanted, expected of a monofocal lens [24]. In another study with data on the same IOL, the TECNIS Monofocal ZCB00, the postoperative VAs were as follows: UDVA of 0.04 ± 0.05 logMAR, a UIVA of 0.19 ± 0.06 logMAR and finally, a UNVA of 1.01 ± 0.68 logMAR [25, 26]. The Intensity outperformed the monofocal lens, especially with the UIVA and UNVA.

Regarding defocus curve, the Intensity diffractive IOL showed that across the curve, the VA achieved was functional and acceptable. Within the binocular defocus range of +1.00 to −3.00 VA was maintained at <0.30 logMAR which from other studies is cited to be the limit of good vision [25]. The defocus curve and CS obtained with this IOL are similar that other multifocal IOLs [27–30]. Compared to the TECNIS Monofocal ZCB00, the defocus curve of the Intensity was rather flat. The defocus curve of the monofocal lens had a steeper shape where from the −1.00 to −3.00 range the logMAR value went above 0.30, indicating poorer vision in the transition to closer visual distances.

An established and valuable part of quantifying image quality involves measuring the ocular wavefront aberrations of an ocular system. The pyramidal wavefront sensor has been a validated method of aberration measurement and been utilized especially in recent years as well as in this study. This new technology measures the said aberrations with higher resolution than other methods [21]. In our study, the retinal optical quality outcomes have been found to be acceptable and stable from results of the higher order aberrations values attained until late into the post-follow-up period. HOA had an RMS of 0.24 µm at 3 months and 0.21 µm at 6 months. At 3 months and 6 months, Trefoil was at 0.11 µm on both follow-ups while coma was 0.10 µm. These values were well below 0.50 µm, which were like reported acceptable mean values for good retinal optical quality [31]. Although for the PSF Strehl ratio (PSFw2), the values were found to be 0.12 ± 0.05 at 3 months and then 0.13 ± 0.05 at 6 months after surgery. Compared to a previous study published concerning various IOLS in relation to retinal image quality [21], the values for the Intensity lens were rather lower for far-distance retinal image quality. However, even with lower values than that of the data resulting from the Intensity that patients’ reported, visual outcomes were not negatively affected, as will be discussed by the PROMs section below. Regardless, the PSFw2 of the Intensity remained stable with no significant change at 3–6 months after surgery, showing that the lens had produced consistent and stable image quality.

The QoV questionnaire given in the postoperative period revealed that although there was presence of photic phenomena, but these were rather mild to moderate and thus tolerated well. Most significant of which were more reported frequent haloes, also with the highest mean values for severity and bothersome attributes, but since they were tolerable, they did not present a significant burden that affected daily life activities. The other two noteworthy subjective symptoms were glare and starbursts but with lower values of their means of frequency, severity, and bothersome features.

In a study that assessed the aberrations of the TECNIS Monofocal ZCB00 [32], the RMS values of the ocular HOAs at a pupil diameter of 4 mm at 3 months were as follows: for total HOA, the RMS was 0.180, for Trefoil it was 0.105, and finally for Coma it was 0.095. The values of the Intensity are similar to the results of a monofocal IOL.

For comparison of the Strehl ratio to monofocal lenses, there were no studies to the authors’ knowledge that were similar to our pupil aperture size of 4 mm. An article published the Strehl ratio of the TECNIS ZCB00 at pupil diameter of 5 mm or larger at 3 months post operation which was found to be 0.28 ± 0.17 [33]. In another study of the TECNIS Monofocal PCB00, the measurement of mean optical quality at 3.0 mm pupil size was 0.23 ± 0.11 while at 5.0 mm aperture, the mean was lesser at 0.16 ± 0.06 [34]. The Intensity produced a ratio of 0.12 ± 0.05 at 3 months, which can be deduced to be inferior to both monofocal IOLs mentioned since even at a smaller aperture, where optical quality may be better, the ratio value was found to be smaller.

Specifically mentioned from the results of the NAVQ-10 was the quality of near vision, where patients had more difficulty seeing close objects in poor or dim light/mesopic conditions and maintaining focus for prolonged near work. The former is expected since diffractive lenses in general cause loss of light in transition areas [1]. Fortunately, patients only reported little difficulty with this phenomenon. With the issue of maintaining focus for prolonged work, factors such as undiagnosed or newly developed dry eye syndrome could be a factor to consider. In totality, most of the patients (90%) had no or little difficulty with these issues with 90% being completely or very satisfied. The PROMs regarding spectacle independence reported that for each distance of VA, 90% were completely independent.

Finally, similar to previous publication [35] regarding the multifocal IOL SeeLens MF, we did not note any significant posterior capsule opacification that necessitated the use of an Nd:YAG laser during the course of the study. The PCO should be evaluated in a longer follow-up period to consolidate these outcomes.

Limitations of this study include the lack of masking and the absence of a control group, which affects the ability to differentiate the actual effect of the lens from a placebo effect or natural variability. The study also has a small sample size, affecting the generalizability of the findings. Our brief observation period may not capture long-term effects or complications associated with the lens, although, with lens studies and follow-ups, 6 months provides a long-term picture of the performance. Furthermore, the applicability of our results is restricted to the specific diopter range of this multifocal IOL, though this range is generally suitable for this type of lens. For cases outside this range, such as with high myopes, implantation of this. IOL would not be considered.

In conclusion, the Intensity IOL successfully restored all range of vision in cataract surgery patients. The relatively flat profile of the defocus curve confirms the five-foci distribution principle of the IOL which provides significant VA from near to distance vision, without compromising far vision and while increasing the depth of focus. The ideal patient profile for the Intensity lens is an individual who prioritizes continuous reading and intermediate-range vision with excellent far vision while being able to tolerate moderate levels of dysphotopsias. The results of this study may contribute to the understanding of the Intensity lens’s performance and help guide clinicians in selecting appropriate intraocular lenses for their patients.

## Summary

### What is known about this topic

- Multifocal IOLs provide vision for all distances affording spectacle independence to patients.
- Due to designs, multifocal IOLs come with a price of side effects that may affect the QoV.
- The type of IOL design depends on patient needs. Surgeon experience and knowledge about IOLs guides prudent utilization.

### What this study adds

- The new design of this IOL provides another option for patients seeking high levels of near-vision spectacle independence.
- Patients were highly satisfied with the implantation of this lens since it provided minimal photic phenomena together with excellent near vision.
- There was preservation of the posterior capsule during the follow-up of the study, not necessitating the need for Nd:YAG Capsulotomy.

## Supplementary information

supplementary information

supplemental material - Figure A

supplemental material - Figure B

eye-reporting-checklist

## Data Availability

The data that support the findings of this study are not openly available due to reasons of sensitivity and are available from the corresponding author upon reasonable request. Data are located in controlled access data storage at Vissum Grupo Miranza and Miranza IOA.
