# Correction: Clinical outcomes with a new diffractive multifocal intraocular lens optimized by the dynamic light utilization algorithm

**DOI:** 10.1038/s41433-024-03495-2

**Published:** 2024-12-02

**Authors:** Jorge L. Alió, Elinor Megiddo Barnir, Ronald Steven S. Medalle, Ana B. Plaza-Puche, Antonio Martínez, Pilar Yébana, Blanca Poyales, Francisco Poyales

**Affiliations:** 1 Vissum Grupo Miranza, Alicante, Spain; 2 Division of Ophthalmology, Universidad Miguel Hernández, Alicante, Spain; 3 Ophthalmology Department, The Edith Wolfson Medical Center, Holon, Israel; 4 Sackler Faculty of Medicine Tel Aviv University, Tel Aviv, Israel; 5 Cornea and Refractive Service, Associated Cebu Eye Specialists, Cebu, Philippines; 6 Department of Ophthalmology, Cebu Institute of Medicine, Cebu, Philippines; 7 Miranza IOA, Madrid, Spain

**Keywords:** Medical research, Diseases, Eye diseases

Correction to: *Eye* 10.1038/s41433-024-03435-0, published online 06 November 2024

The article “Clinical outcomes with a new diffractive multifocal intraocular lens optimized by the dynamic light utilization algorithm”, written by Jorge L. Alió, Elinor Megiddo Barnir, Ronald Steven S. Medalle II, Ana B. Plaza-Puche, Antonio Martínez, Pilar Yébana, Blanca Poyales and Francisco Poyales, was originally published electronically on the publisher’s internet portal on 6 November 2024 without open access. With the author(s)’ decision to opt for Open Choice the copyright of the article changed on 14 November 2024 to © The Author(s) 2024 and the article is forthwith distributed under a Creative Commons Attribution 4.0 International License, which permits use, sharing, adaptation, distribution and reproduction in any medium or format, as long as you give appropriate credit to the original author(s) and the source, provide a link to the Creative Commons licence, and indicate if changes were made.

The images or other third party material in this article are included in the article’s Creative Commons licence, unless indicated otherwise in a credit line to the material. If material is not included in the article’s Creative Commons licence and your intended use is not permitted by statutory regulation or exceeds the permitted use, you will need to obtain permission directly from the copyright holder.

To view a copy of this licence, visit http://creativecommons.org/ licenses/by/4.0/.

