# Supplementary material for: Clinical outcomes with a new diffractive multifocal intraocular lens optimized by the dynamic light utilization algorithm
Source: Eye (Lond). 2024 Nov 6;39(2):359–65. doi: 10.1038/s41433-024-03435-0 (PMC11751119; doi:10.1038/s41433-024-03435-0)
Supplement: Supplementary file 1 — supplementary information [file 41433_2024_3435_MOESM1_ESM.docx]

**Supplementary Information**

Figure A Intensity IOL: Image of the Intensity IOL

Figure B. Mean postoperative contrast sensitivity function (CSF) in patients implanted with the Intensity MF IOL in logarithmic scale under mesopic conditions at 1 month and 6 months postoperatively
