# Supplementary figures and images for: Clinical outcomes with a new diffractive multifocal intraocular lens optimized by the dynamic light utilization algorithm
Source: Eye (Lond). 2024 Nov 6;39(2):359–65. doi: 10.1038/s41433-024-03435-0 (PMC11751119; doi:10.1038/s41433-024-03435-0)

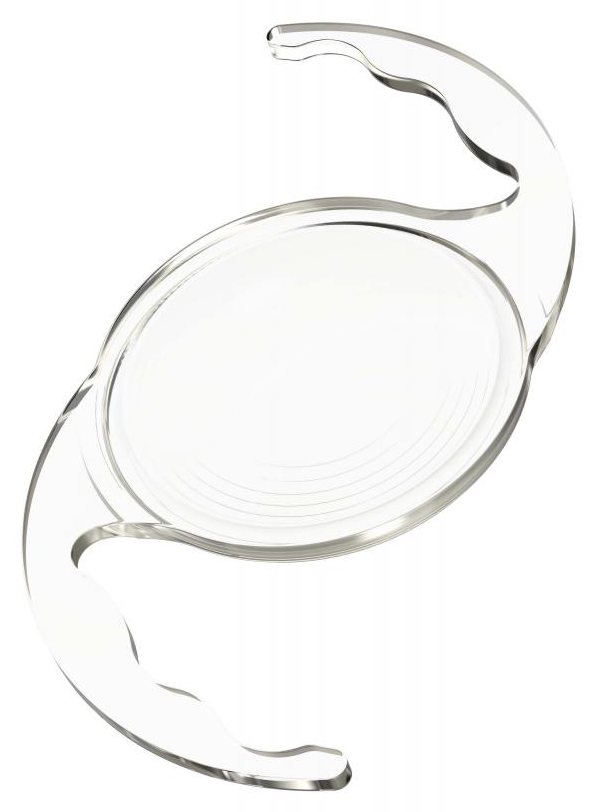

Supplement: Supplementary file 2 — supplemental material - Figure A [file 41433_2024_3435_MOESM2_ESM.tif]

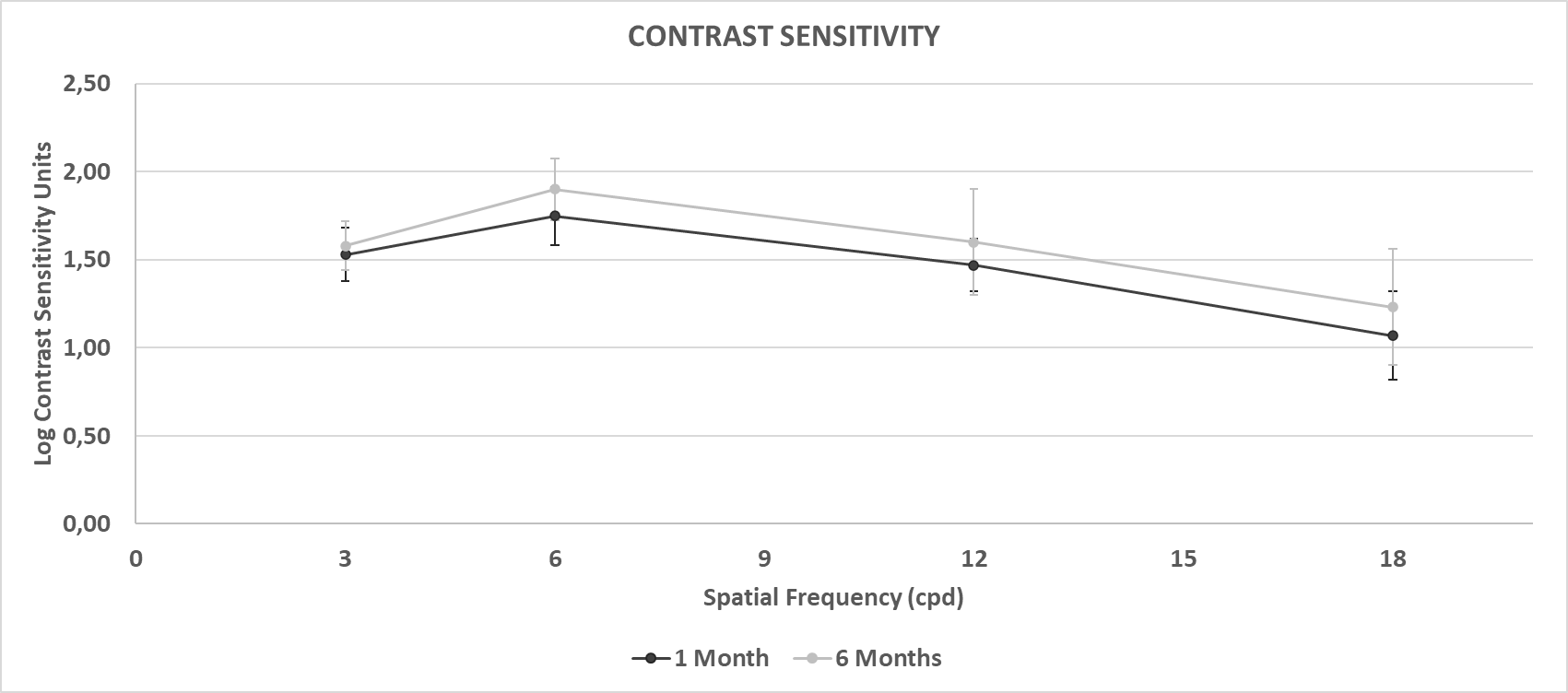

Supplement: Supplementary file 3 — supplemental material - Figure B [file 41433_2024_3435_MOESM3_ESM.tif]
